# Nonlinear Electrical Conductivity and Thermal Conductivity of g-C_3_N_4_/Liquid Silicone Rubber Field Grading Composites

**DOI:** 10.3390/ma19112367

**Published:** 2026-06-02

**Authors:** Peng Han, Jiayang Li, Peng Hu, Zheng Zhang, Chen Zhao, Dongli Zhang

**Affiliations:** 1 School of Materials Science and Engineering, North China University of Water Resources and Electric Power, Zhengzhou 450045, China; 2 School of Electrical and Information Engineering, Zhengzhou University of Light Industry, Zhengzhou 450002, China; 3 Academy of Agricultural Planning and Engineering, Key Laboratory of Technologies and Models for Cyclic Utilization from Agricultural Resources, Ministry of Agriculture and Rural Affairs, Beijing 100125, China

**Keywords:** graphitic carbon nitride, oxygen doping, nonlinear electrical conductivity, thermal conductivity, field grading composite

## Abstract

The uneven electric field in cable accessory insulation can be optimized by field grading composite (FGC). We explored graphitic carbon nitride (g-C_3_N_4_) as a filler in liquid silicone rubber (LSR) matrices. Oxygen-doped g-C_3_N_4_ (O-g-C_3_N_4_) was prepared via calcination of g-C_3_N_4_ with ascorbic acid. Composites of g-C_3_N_4_/LSR and O-g-C_3_N_4_/LSR with different filler contents were fabricated. Microstructural and optical characterizations demonstrate that O-g-C_3_N_4_ retains the crystal structure of pristine g-C_3_N_4_ but exhibits thinner layers, modified elemental composition, and a 27.8% reduction in band gap; fillers are uniformly dispersed in the LSR matrix. Experimental measurements reveal that both composites exhibit nonlinear conductivity, while O-g-C_3_N_4_/LSR shows more pronounced nonlinearity at lower filler contents, accompanied by a faster decline in dielectric breakdown strength. There is little difference in thermal conductivity between g-C_3_N_4_/LSR and O-g-C_3_N_4_/LSR composites with the same filler content, which indicates that the change in band gap width has no significant influence on thermal conductivity. The low-cost synthesis and simple bandgap tuning method of g-C_3_N_4_ provide certain advantages for its use as a nonlinear filler in the preparation of FGC, broadening the application fields of g-C_3_N_4_, and verifying the possibility of reducing FGC filler usage through bandgap tuning.

## 1. Introduction

As critical yet vulnerable components in power transmission systems, cable accessories operate for extended periods in complex environments characterized by multi-physical field coupling, leading to a relatively high failure rate—with insulation failures accounting for the majority of cases [1,2,3,4,5]. Field grading composite (FGC) is essential for high-voltage cable accessories. Their nonlinear conductivity enables the homogenization of uneven electric fields within cable accessories, making them a focus of intensive research [6,7,8,9,10]. It is widely accepted that the formation of double Schottky barriers or semiconductor junctions is responsible for the nonlinear conductivity of such composites, thereby enabling electric field grading.

FGC typically consists of an insulating matrix and semiconductive fillers. Commonly used matrices include silicone rubber (SR), ethylene propylene diene monomer (EPDM), and epoxy resin (ER). For semiconductive fillers, researchers primarily utilized carbon black (CB), silicon carbide (SiC) and zinc oxide (ZnO) [8]. They are widely used in the production of cable accessories, primarily because their prices are relatively low, especially for CB, while still meeting the voltage level requirements. However, exploring new applications for materials remains highly necessary. Building on this understanding, researchers have explored various alternative materials as fillers, including CCTO (CaCu_3_Ti_4_O_12_) nanoparticles and nanofibers [11], TiO_2_ [12,13], and our previous series of studies on WS_2_ [14,15,16]. Given the typical service conditions of FGC, enhancing their thermal conductivity is recognized as a key factor for expanding their practical applications [17,18,19,20,21,22,23,24,25]. Meanwhile, the low cost of raw materials also makes it have certain potential for application.

Graphitic carbon nitride (g-C_3_N_4_) is a versatile photocatalyst with a band gap of approximately 2.7 eV and a thermal conductivity of approximately 120 W/(m·K). It has been extensively used in applications such as photocatalytic degradation of pollutants [26] and photocatalytic water splitting for hydrogen production [27,28]. Considering its two-dimensional (2D) crystalline structure and tunable semiconductive properties, g-C_3_N_4_ holds potential as a nonlinear conductive filler for FGC [29,30].

In this work, g-C_3_N_4_ was prepared via a facile method, and oxygen doping was performed to modulate its band gap [30]. LSR was used as the matrix to fabricate g-C_3_N_4_/LSR and O-g-C_3_N_4_/LSR composites. Microscopic characterization techniques, including SEM, TEM, XRD, XPS, and UV-Vis DRS, were employed to investigate the physical properties of the semiconductive fillers and composites. Subsequently, the DC conductivity, dielectric breakdown strength, and thermal conductivity of FGC were tested and discussed.

## 2. Fabrication of Composites

### 2.1. Raw Materials

The raw materials employed in this experiment are specified in Table 1.

### 2.2. Preparation of g-C_3_N_4_

A suitable amount of melamine was placed in a covered crucible, which was then transferred to a muffle furnace (NBD-M1500-22IT, NOBODY Materials Science & Technology Co., Ltd., Zhengzhou, China). The furnace was heated at a rate of 2 °C/min to 550 °C and held at this temperature for 4 h. After natural cooling to room temperature, the resulting yellow powder was identified as graphitic carbon nitride (g-C_3_N_4_).

### 2.3. Preparation of O-g-C_3_N_4_

A total of 10 g of the as-prepared g-C_3_N_4_ and 5 g of Vc were mixed in an agate mortar and ground for 60 min. The mixture was then transferred to a covered crucible, which was placed in a muffle furnace. The furnace was heated at a rate of 2 °C/min to 300 °C and maintained for 2 h. After the muffle furnace cooled naturally to room temperature, the obtained powder was confirmed as O-g-C_3_N_4_.

### 2.4. Preparation of g-C_3_N_4_/LSR and O-g-C_3_N_4_/LSR Composites

We adopted parts per hundred rubber/resin (phr) as the unit of measurement, with the corresponding weight fractions tabulated in Table 2. This choice was made for two primary reasons. First, to facilitate composite production coordination with enterprises. Second, to ensure consistent comparability and continuity with our previous research. Additionally, the use of phr offers a distinct advantage. As filler loading increases, the incremental interval between different filler content levels is narrower than that when using mass percentage, which facilitates more precise control over filler dosage in sample preparation.

The preparation process of g-C_3_N_4_/LSR and O-g-C_3_N_4_/LSR composites is identical. We took g-C_3_N_4_ as an example filler. First, g-C_3_N_4_ was incorporated into Component A of LSR, and the mixture was then thoroughly mixed with Component B of LSR to prepare g-C_3_N_4_/LSR composites with g-C_3_N_4_ loadings ranging from 10 to 60 phr. Subsequently, the mixture was subjected to hot-press vulcanization using a plate vulcanizer (25t-350 × 350, Zhengzhou Huanke Machinery Manufacturing Co., Ltd., Zhenghzou, China) under the conditions of 130 °C and 15 MPa for 10 min. The obtained sheet samples were further post-cured in an oven at 100 °C for 12 h to ensure complete vulcanization, and finally, g-C_3_N_4_/LSR composites with g-C_3_N_4_ contents of 10–60 phr were successfully obtained.

## 3. Experimental Characterization

### 3.1. Microstructure Characterization

The dispersion of fillers in the composites was observed using a tungsten filament scanning electron microscope (SEM, VEGA 3 LMU, TESCAN, Brno, Czech Republic). The microscopic structure characterization of g-C_3_N_4_ and O-g-C_3_N_4_ was observed using transmission electron microscopy (TEM, JEM-F200, JEOL, Tokyo, Japan). The crystal phase of the fillers was analyzed via an X-ray diffractometer (XRD, DX-2700, Haoyuan Instrument Co., Ltd., Dandong, China). The surface chemical composition and chemical bonding states of the fillers were determined using an X-ray photoelectron spectrometer (XPS, K-alpha, Thermo Fisher Scientific, Waltham, MA, USA). UV-visible diffuse reflectance spectroscopy (UV-Vis DRS, UH4150, Hitachi, Tokyo, Japan) was used to determine the absorbance of g-C_3_N_4_ and O-g-C_3_N_4_ at different wavelengths, which will be used to calculate the bandgap widths.

### 3.2. DC Conductivity

The DC conductivity measurement system was identical to that used in our previous work [14,15,16], consisting of a Keithley 2635B microammeter, a Keithley Model 2290 digital high-voltage source (Solon, OH, USA), and a self-made temperature-controlled box. Data acquisition was performed using a self-programming program. Prior to the measurement, all samples were clamped between electrodes in the shielded oven and equilibrated for 30 min after the temperature stabilized. The thickness of the samples was approximately 200 μm. The test electric field strength ranged from 1 kV/mm to 20 kV/mm, with a step size of 1 kV/mm, and the current was recorded every 15 s.

### 3.3. Dielectric Property

The dielectric properties of the composites were measured using a broadband dielectric spectrometer (Concept40, Novocontrol GmbH, Montabaur, Germany) at room temperature over a frequency range of 1 Hz to 10^5^ Hz. Prior to testing, both surfaces of the samples were sputtered with gold using magnetron sputtering equipment (ETD-900M, Beijing Elaborate Technology Development Ltd., Beijing, China) to ensure good electrical contact.

### 3.4. Dielectric Breakdown Strength

The dielectric breakdown strength of the composites was measured using a dielectric breakdown tester (BDJC-50KV, Beijing Beiguang Jingyi Instrument Equipment Co., Ltd., Beijing, China) at room temperature, with a voltage ramp rate of 200 V/s.

### 3.5. Thermal Conductivity

The thermal conductivity (*λ*) of the composites was calculated using the following formula:

(1) λ=α⋅ρ⋅C

where *λ* is the thermal conductivity (W/(m·K)), *α* is the thermal diffusivity (m^2^/s), *ρ* is the density (kg/m^3^), and C is the specific heat capacity (J/(kg·K)).

The thermal diffusivity (*α*) was measured using a laser flash diffusivity instrument (LFA 467 HyperFlash, NETZSCH, Selb, Germany). Prior to measurement, the upper and lower surfaces of the samples (12.7 mm in diameter, 0.9 mm in thickness) were uniformly sprayed with ultrafine graphite powder (GRAPHIT 33 Spray, Kontakt Chemie, CRC Industries, Iffezheim, Germany) to enhance laser absorption. The density (*ρ*) of the samples was determined via the weighing method. The specific heat capacity (*C*) was measured using a differential scanning calorimeter (DSC-60, Shimadzu, Kyoto, Japan) at a heating rate of 10 °C/min, in accordance with the standard [31].

## 4. Results and Discussion

### 4.1. Microscopic Characterization

**Figure 1:**
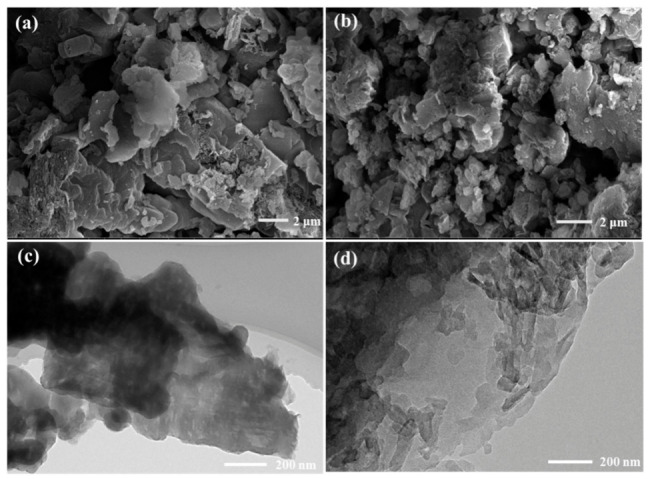
SEM and TEM images of g-C_3_N_4_ and O-g-C_3_N_4_. (**a**) SEM of g-C_3_N_4_; (**b**) SEM of O-g-C_3_N_4_; (**c**) TEM of g-C_3_N_4_; (**d**) TEM of O-g-C_3_N_4_.

SEM images of g-C_3_N_4_ and O-g-C_3_N_4_, as can be seen in Figure 1a,b, reveal that both materials exhibit a stacked layered structure with essentially identical morphologies. TEM images, as shown in Figure 1c,d, demonstrate that the layers of O-g-C_3_N_4_ are significantly thinner than those of g-C_3_N_4_. The average sheet size of g-C_3_N_4_ is about 350 nm. The average sheet size of O-g-C_3_N_4_ is about 280 nm. O-g-C_3_N_4_ shows smaller lamellar size and weaker agglomeration due to exfoliation during oxygen doping.

This phenomenon can be attributed to the substitution of partial N atoms in g-C_3_N_4_ by O atoms, which disrupts the original planar π-π conjugated network of g-C_3_N_4_. Additionally, the introduction of O atoms weakens the van der Waals forces between g-C_3_N_4_ layers, facilitating the exfoliation of individual layers from the bulk material.

**Figure 2:**
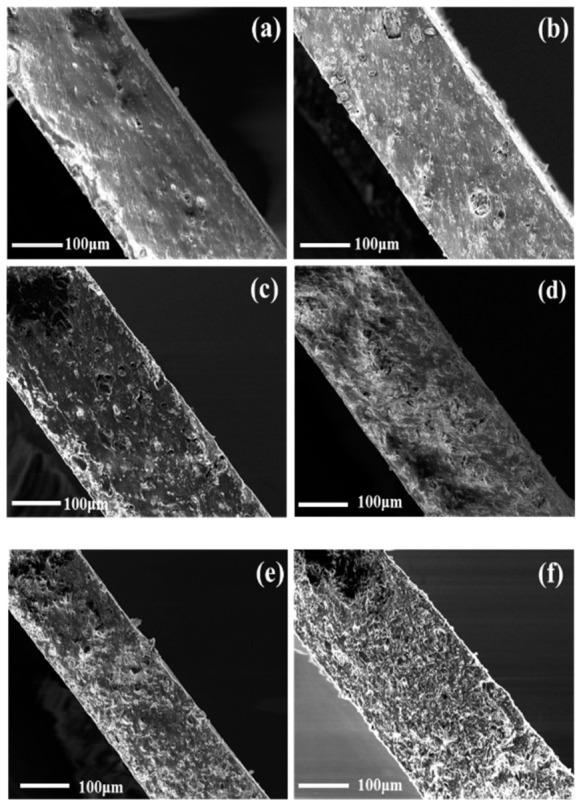
Cross-sectional SEM images of g-C_3_N_4_/LSR composites with different filler contents: (**a**) 10 phr; (**b**) 20 phr; (**c**) 30 phr; (**d**) 40 phr; (**e**) 50 phr; (**f**) 60 phr.

**Figure 3:**
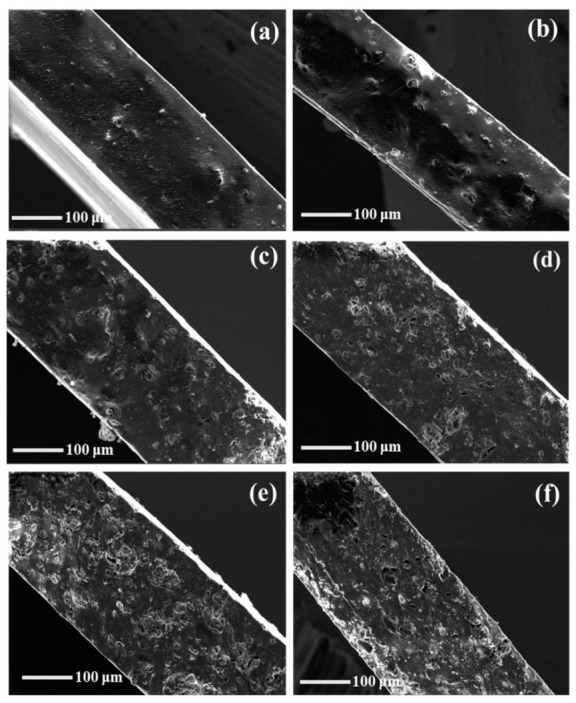
Cross-sectional SEM images of O-g-C_3_N_4_/LSR composites with different filler contents. (**a**) 10 phr; (**b**) 20 phr; (**c**) 30 phr; (**d**) 40 phr; (**e**) 50 phr; (**f**) 60 phr.

Figure 2 and Figure 3 present cross-sectional SEM images of g-C_3_N_4_/LSR and O-g-C_3_N_4_/LSR composites with filler contents of 10–60 phr. As observed from these figures, the dispersion of g-C_3_N_4_ in g-C_3_N_4_/LSR composites is similar to that of O-g-C_3_N_4_ in O-g-C_3_N_4_/LSR composites. For composites with 10 phr and 20 phr fillers, only sporadic filler particles are visible on the cross-sections, with large interparticle distances. As the filler content increases, particle aggregation becomes increasingly prominent: at 40 phr, most filler particles are in contact with each other; at 60 phr, the fillers exhibit a dense distribution.

### 4.2. XRD Analysis

**Figure 4:**
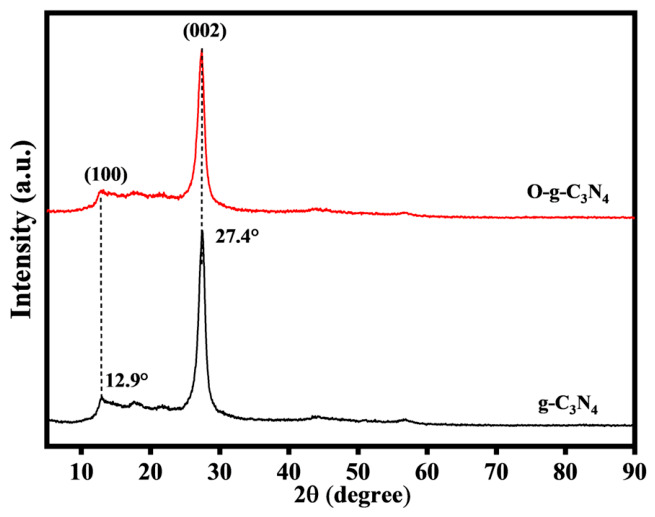
XRD patterns of g-C_3_N_4_ and O-g-C_3_N_4_.

Figure 4 shows the XRD patterns of g-C_3_N_4_ and O-g-C_3_N_4_. The diffraction profiles of O-g-C_3_N_4_ are similar to those of pristine g-C_3_N_4_, with characteristic diffraction peaks at 12.9° (corresponding to the (100) crystal plane) and 27.4° (corresponding to the (002) crystal plane)—both of which are typical of the g-C_3_N_4_ structure [29]. This indicates that oxygen doping does not destroy the basic crystal structure of g-C_3_N_4_.

Compared with g-C_3_N_4_, O-g-C_3_N_4_ shows reduced diffraction intensity and slightly increased full width at half maximum (FWHM), indicating increased structural disorder and defect formation after oxygen doping. Calculations were performed to support these conclusions. According to Bragg’s law:

(2) 2dsin θ=nλ

where *d* represents the interplanar spacing, θ denotes the angle between the incident X-ray and the corresponding crystal plane, *λ* signifies the wavelength of the X-ray (Cu target XRD standard wavelength *λ* = 0.15406 nm), and *n* stands for the diffraction order. To ensure reliability in the calculation, both the (100) and (002) crystal planes were used for interlayer spacing evaluation, instead of a single diffraction peak.

(3) $$g\text{-}C_{3}N_{4}:d_{\left(002\right)}=0.326\;nm$$

(4) $$O\text{-}g\text{-}C_{3}N_{4}:d_{\left(002\right)}=0.321\;nm$$

(5) $$g\text{-}C_{3}N_{4}:d_{\left(100\right)}=0.686\;nm$$

(6) $$O\text{-}g\text{-}C_{3}N_{4}:d_{\left(100\right)}=0.682\;nm$$

Using the Scherrer formula:

(7) D=K⋅λ/(β⋅cos θ)

where K is the Scherrer constant, λ is the X-ray wavelength, β is the full width at half maximum of the diffraction peak (based on the measured FWHM of typical g-C_3_N_3_), and θ is the Bragg diffraction angle. Notably, instrumental broadening correction was performed on the raw measured FWHM prior to calculation to eliminate the inherent broadening effect of the XRD instrument and improve the credibility of crystallite size results. The crystallite sizes of g-C_3_N_4_ and O-g-C_3_N_4_ along the (002) plane were 7.72 nm and 6.15 nm, and those along the (100) plane were 9.86 nm and 8.23 nm, respectively.

These quantitative results directly verify that oxygen doping reduces interlayer spacing and crystallite size, introducing structural defects while preserving the main g-C_3_N_4_ crystal structure.

The reduced interlayer spacing and increased defects provide more carrier transport channels and improve nonlinear conductivity. They introduce more interfaces and reduce breakdown strength. They slightly affect phonon scattering but have little influence on thermal conductivity. This is added in Section 4.2.

### 4.3. XPS Analysis

**Figure 5:**
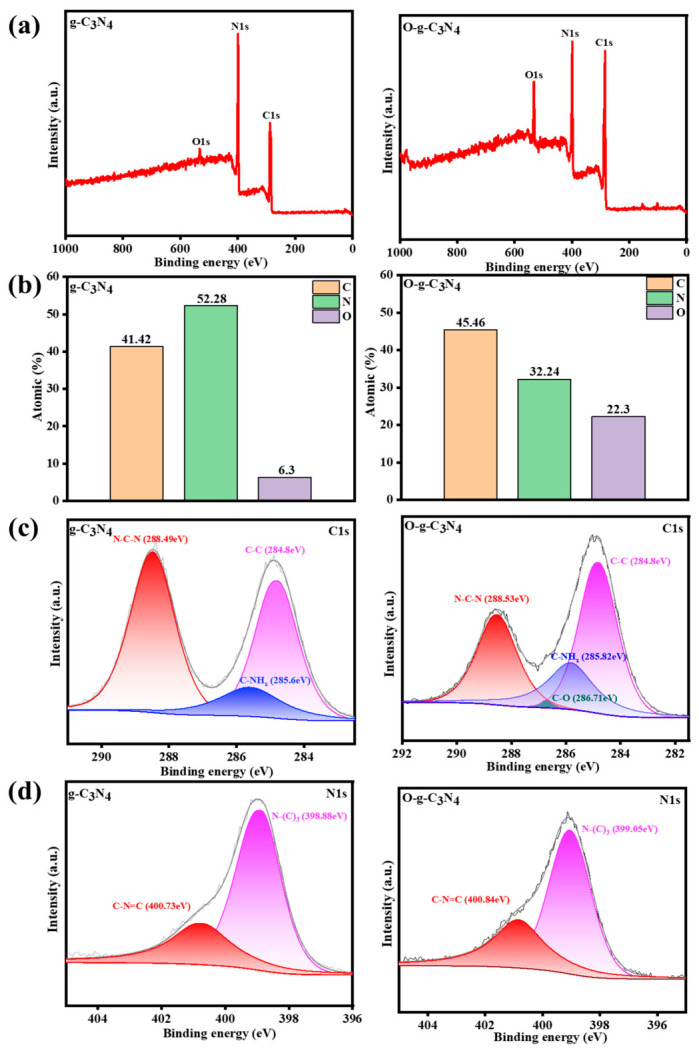

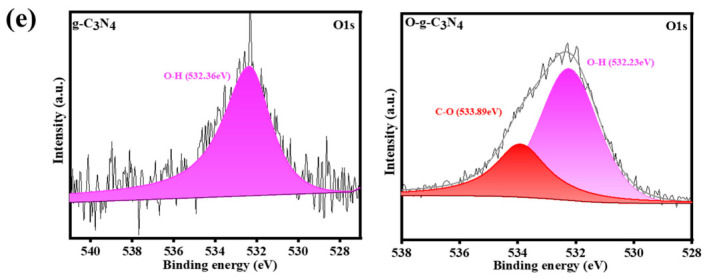
XPS analysis of g-C_3_N_4_ and O-g-C_3_N_4_. (**a**) survey spectra; (**b**) elemental composition (C, N, O); (**c**) high-resolution C 1s spectra; (**d**) high-resolution N 1s spectra; (**e**) high-resolution O 1s spectra.

The XPS spectra of g-C_3_N_4_ and O-g-C_3_N_4_ are presented in Figure 5. As shown in Figure 5a, both samples exhibit three characteristic peaks corresponding to C 1s, N 1s, and O 1s. Figure 5b indicates that the oxygen content of O-g-C_3_N_4_ (22.3 wt%) is significantly higher than that of pristine g-C_3_N_4_ (6.3 wt%), while its nitrogen content (32.24 wt%) is lower than that of g-C_3_N_4_ (52.28 wt%). It should be emphasized that XPS is a surface-sensitive semi-quantitative technique, and the obtained oxygen content of 22.3 wt% only reflects the surface relative composition rather than the bulk absolute doping level. Therefore, the reported oxygen content of 22.3 wt% is regarded as a relative surface atomic ratio rather than an absolute quantitative value. Due to the similar ionic radius and electronegativity between O and N atoms, oxygen can partially substitute nitrogen sites in the g-C_3_N_4_ lattice. The formed nitrogen vacancies and structural defects provide abundant active sites for oxygen adsorption and bonding, which enables a relatively high oxygen content on the material surface without destroying the overall valence balance and crystal structure. The formation of nitrogen vacancies and structural defects further promotes the high-level oxygen doping, which fundamentally realizes the high doping concentration of O in the g-C_3_N_4_ framework.

In the high-resolution C 1s spectra, as shown in Figure 5c, the peaks at 284.8 eV, 285.7 eV, and 288.5 eV correspond to adventitious carbon, sp^2^-hybridized carbon in the -NH_2_ groups on the aromatic ring, and carbon in the N–C=N bond, respectively. Notably, O-g-C_3_N_4_ exhibits a new peak at 286.71 eV, which is attributed to the C-O bond—further confirming the successful oxygen doping of g-C_3_N_4_.

In the high-resolution N 1s spectra, as shown in Figure 5d, the peaks at 398.9 eV and 400.8 eV correspond to N-(C)_3_ and C–N=C groups in the triazine unit, respectively [16]. In the high-resolution O 1s spectra, as shown in Figure 5e, the peak at 532.3 eV is assigned to the O-H bond. O-g-C_3_N_4_ shows an additional peak at 533.89 eV, which is attributed to the C-O bond. This indicates that oxygen exists not only in the form of O-H groups on the surface of g-C_3_N_4_ but also in the bulk structure in the form of C-O bonds—confirming the successful doping of oxygen into the g-C_3_N_4_ lattice [30].

### 4.4. UV-Vis DRS

**Figure 6:**
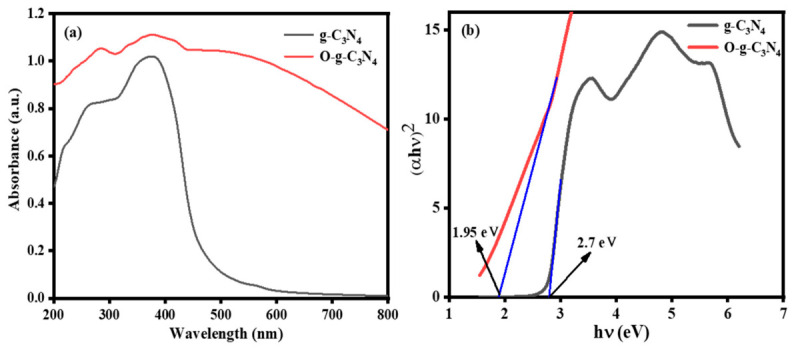
(**a**) UV-Vis DRS diagram of g-C_3_N_4_ and O-g-C_3_N_4_; (**b**) g-C_3_N_4_ and O-g-C_3_N_4_ band gaps calculated by the Tauc method.

UV-visible diffuse reflectance spectroscopy (UV-Vis DRS) was used to determine the absorbance of g-C_3_N_4_ and O-g-C_3_N_4_ at different wavelengths, as shown in Figure 6a. It is evident that O-g-C_3_N_4_ exhibits higher absorbance than g-C_3_N_4_ across the entire spectral range. The band gaps of the samples were calculated using the Tauc method, as shown in Figure 6b, yielding values of 2.7 eV for g-C_3_N_4_ and 1.95 eV for O-g-C_3_N_4_. This represents a 27.8% reduction in band gap after oxygen doping. The linear region near the absorption edge was clearly selected and marked for band gap extrapolation. The linear fitting range was determined by ignoring the low-energy tail absorption and only adopting the valid high-absorption linear segment to ensure the accurate estimation of the optical band gap.

The band gap was calculated using the Tauc equation:

(8) $${\left(\alpha h\nu \right)}^{1/2}=A(h\nu -Eg)$$

where α is the absorption coefficient, h is Planck’s constant, ν is photon frequency, A is constant, and Eg is the optical band gap. The curve was plotted with (αhν)^1/2^ vs. hν, and Eg was obtained by extrapolating the linear region to the horizontal axis.

The reduction in band gap can be attributed to the introduction of intrinsic defect levels within the band gap of g-C_3_N_4_ induced by oxygen doping.

Combined with the above XRD and XPS results, oxygen doping introduces lattice distortion, nitrogen vacancies, and structural defects in the g-C_3_N_4_ framework, and partial substitution of nitrogen by oxygen atoms changes the local electronic coordination environment. These intrinsic structural and chemical defects inevitably generate intermediate defect levels within the intrinsic band gap of g-C_3_N_4_, which serve as transitional states for electron transitions. As a result, the required photoexcitation energy is reduced, leading to the obvious narrowing of the optical band gap of O-g-C_3_N_4_. The variation trend of optical absorption obtained from UV-Vis DRS further supports this defect-level-induced bandgap-narrowing mechanism.

### 4.5. Electrical Conductivity

**Figure 7:**
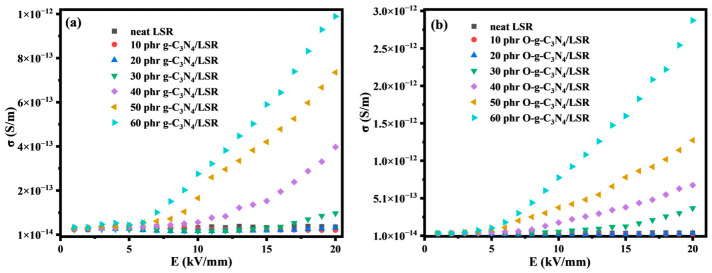
Electrical conductivity of composites with different filler contents. (**a**) g-C_3_N_4_/LSR; (**b**) O-g-C_3_N_4_/LSR.

Figure 7 shows the electrical conductivity of g-C_3_N_4_/LSR and O-g-C_3_N_4_/LSR composites with different filler contents.

For g-C_3_N_4_/LSR composites, as shown in Figure 7a, those with 10–20 phr g-C_3_N_4_ exhibit conductivity similar to that of pure LSR, maintaining good insulating properties and no nonlinear conductivity. At 30 phr, nonlinear conductivity begins to appear at approximately 20 kV/mm. For composites with 40–60 phr g-C_3_N_4_, distinct nonlinear conductivity is observed—with conductivity at 20 kV/mm being approximately two orders of magnitude higher than that at 1 kV/mm.

From a microscopic perspective, when the external electric field strength is below the threshold value, the introduction of fillers and intrinsic material defects creates a large number of traps within the composite. Low-energy charge carriers are easily captured by these traps during migration to the anode, resulting in nearly constant macroscopic conductivity. When the field strength exceeds the threshold, charge carriers gain sufficient energy to escape from the traps and migrate toward the anode—leading to a rapid increase in macroscopic conductivity.

With increasing g-C_3_N_4_ content, the conductivity of the 60 phr composite at 20 kV/mm is nearly three orders of magnitude higher than that at 1 kV/mm. This phenomenon can be attributed to three factors: (1) The narrow band gap of g-C_3_N_4_ (~2.7 eV) facilitates electron transition across the band gap; (2) the semiconductive nature of g-C_3_N_4_ leads to an increase in the number of charge carriers with increasing filler content; (3) the 2D sheet-like structure of g-C_3_N_4_ promotes the formation of conductive paths when the filler content reaches a critical threshold.

As shown in Figure 7b, similar to g-C_3_N_4_/LSR composites, O-g-C_3_N_4_/LSR composites with 10–20 phr fillers maintain good insulation and exhibit no nonlinear conductivity within the test field strength range (1–20 kV/mm). At 30 phr, O-g-C_3_N_4_/LSR composites show significantly more pronounced nonlinear conductivity than g-C_3_N_4_/LSR composites with the same filler content. This is attributed to the 27.8% band gap reduction (from 2.7 eV to 1.95 eV), which lowers the energy barrier for carrier excitation and enables earlier onset of nonlinear conductivity.

The nonlinear coefficient of O-g-C_3_N_4_/LSR composites increases significantly with increasing filler content. At low filler contents, the large interparticle distance prevents O-g-C_3_N_4_ from fully exploiting its band gap reduction advantage—resulting in performance similar to that of g-C_3_N_4_/LSR. As the filler content increases, the interparticle distance decreases. For O-g-C_3_N_4_/LSR composites with a narrower band gap, charge carriers can more easily overcome the potential barrier and form conductive paths under high electric fields—leading to enhanced nonlinear conductivity.

### 4.6. Dielectric Breakdown Strength

**Figure 8:**
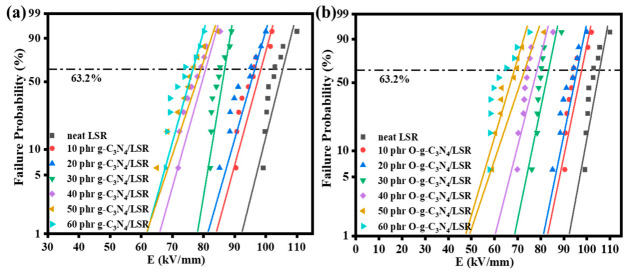
Weibull distribution plots of dielectric breakdown strength of composites. (**a**) g-C_3_N_4_/LSR; (**b**) O-g-C_3_N_4_/LSR.

Figure 8 presents the Weibull distribution plots of the breakdown strength for g-C_3_N_4_/LSR and O-g-C_3_N_4_/LSR composites, and Table 3 lists the Weibull parameters (characteristic breakdown strength E_0_ and shape parameter α) for composites with different filler contents.

Combining Figure 8 and Table 3, the filler content of g-C_3_N_4_ in g-C_3_N_4_/LSR composites exhibits a significant negative correlation with the breakdown strength. For the 10 phr composite, the breakdown strength decreases by approximately 10% compared to pure LSR. With further increases in g-C_3_N_4_ content, the breakdown strength of the composites continues to decrease: when the filler content reaches 60 phr, the breakdown strength decreases from 108.2 kV/mm (pure LSR) to 75.1 kV/mm.

This phenomenon can be explained by two factors. First, the semiconductive nature of g-C_3_N_4_ introduces additional charge carriers into the LSR matrix. Under high electric fields, these carriers tend to move directionally, increasing the risk of dielectric breakdown. Second, high filler loadings introduce structural defects and form filler networks, which further deteriorate the breakdown performance of the composites.

Compared with g-C_3_N_4_/LSR composites, O-g-C_3_N_4_/LSR composites show minimal differences in breakdown strength (less than 1 kV/mm) when the filler content is 10–20 phr. As the filler content increases, the gap in breakdown strength between the two types of composites gradually widens. For the 60 phr, the breakdown strength of O-g-C_3_N_4_/LSR is 9.1 kV/mm lower than that of g-C_3_N_4_/LSR.

### 4.7. Thermal Conductivity

**Figure 9:**
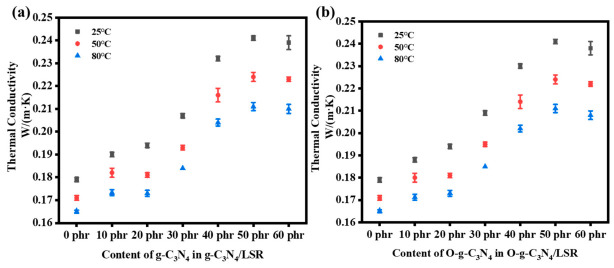
Thermal conductivity of composites with different filler contents. (**a**) g-C_3_N_4_/LSR; (**b**) O-g-C_3_N_4_/LSR.

Figure 9 shows the thermal conductivity of g-C_3_N_4_/LSR and O-g-C_3_N_4_/LSR composites with different filler contents, measured at 25 °C, 50 °C, and 80 °C.

As illustrated in Figure 9, the thermal conductivity of pure LSR at 25 °C is only 0.179 W/(m·K). With increasing g-C_3_N_4_ content, the thermal conductivity of g-C_3_N_4_/LSR composites initially increases. At 50 phr, the thermal conductivity reaches 0.241 W/(m·K), representing a 35% enhancement compared to pure LSR. However, further increases in g-C_3_N_4_ content lead to a decrease in thermal conductivity.

This trend can be attributed to the following mechanism: At low filler contents, increasing g-C_3_N_4_ loading facilitates the formation of thermal conduction networks. Since the thermal conductivity of g-C_3_N_4_ is higher than that of LSR, heat can be rapidly transferred through these networks via phonon scattering—resulting in improved thermal conductivity. At high filler contents, excessive g-C_3_N_4_ particles agglomerate, which disrupts phonon scattering and reduces heat transfer efficiency.

Notably, the thermal conductivity trends of g-C_3_N_4_/LSR and O-g-C_3_N_4_/LSR composites at 50 °C and 80 °C are consistent with those at 25 °C. However, under the same filler content, the thermal conductivity of the composites decreases with increasing temperature. This is because higher temperatures reduce the mean free path of phonons and increase phonon collision frequency—weakening the effective scattering and transfer of phonons, thereby lowering thermal conductivity.

Similar to g-C_3_N_4_/LSR composites, the thermal conductivity of O-g-C_3_N_4_/LSR composites shows no significant difference from that of g-C_3_N_4_/LSR, but increases gradually with filler content and peaks at 50 phr. This aligns with the general understanding that an optimal filler content maximizes the thermal performance of composites. The negligible difference in thermal conductivity between O-g-C_3_N_4_/LSR and g-C_3_N_4_/LSR composites indicates that phonon scattering and heat transfer are primarily influenced by temperature and material structure, rather than band gap width.

## 5. Conclusions

The comprehensive structure–property relationship can be established as follows. Structural characterizations from XRD and XPS confirm that oxygen doping introduces more structural defects and reduces the crystallite size and interlayer spacing of g-C_3_N_4_. These defects and modified chemical states lead to a narrowed band gap as verified by UV-Vis DRS, which effectively lowers the energy barrier for carrier excitation and thus significantly enhances the nonlinear electrical conductivity of the composite. Meanwhile, morphological observations from SEM and TEM reveal that O-g-C_3_N_4_ possesses thinner and smaller nanosheets than pure g-C_3_N_4_, which favors better dispersion in the LSR matrix and promotes earlier formation of conductive paths under electric fields. Furthermore, the changes in layer spacing and defect density mainly affect carrier transport and electrical behavior, while exerting negligible influence on phonon transport and thermal conductivity, which is consistent with the measured thermal performance of the composites.

In summary, oxygen doping of g-C_3_N_4_ results in about a 27.8% reduction in band gap width compared to pristine g-C_3_N_4_, without destroying its basic crystal structure. Performance tests on composites demonstrate that oxygen doping significantly enhances the electric field regulation capability of g-C_3_N_4_/LSR composites—particularly at a filler content of 30 phr, where O-g-C_3_N_4_/LSR exhibits more pronounced nonlinear conductivity than g-C_3_N_4_/LSR. However, oxygen doping also leads to a faster decline in the breakdown strength of the composites, especially at high filler contents. In terms of thermal conductivity, both g-C_3_N_4_/LSR and O-g-C_3_N_4_/LSR composites show similar trends: thermal conductivity increases with filler content up to 50 phr (35% enhancement vs. pure LSR) and then decreases, with no significant difference between the two composite systems.

## Figures and Tables

**Table 1:** Basic Information of Raw Materials and Reagents.

Raw Materials and Reagents	Chemical Formula	Purity	Manufacturer
Melamine	[C_3_N_3_(NH_2_)_3_]	Analytical grade	Tianjin Damao Chemical Reagent Co., Ltd., Tianjin, China
L(+)-Ascorbic Acid (Vc)	C_6_H_8_O_6_	Analytical grade	Xilong Scientific Co., Ltd., Guangzhou, China
Absolute Ethanol	C_2_H_6_O	Analytical grade	Tianjin Hengxing Chemical Reagent Co., Ltd., Tianjin, China
Deionized Water	H_2_O	Analytical grade	Laboratory-made
Liquid Silicone Rubber (LSR) POWERSIL 745AB	((−CH_3_)_2_SiO)_n_	--	Wacker Chemie AG, Munich, Germany

**Table 2:** Conversions of filler content in different units.

Counting Unit	Filler Content					
Dosage in phr	10	20	30	40	50	60
Weight fraction (wt%)	9.09	16.67	23.08	28.57	33.33	37.5

**Table 3:** Weibull distribution parameters of g-C_3_N_4_/LSR and O-g-C_3_N_4_/LSR composites.

Composite Type	Parameters of Weibull Distribution		
	Filler Contents	E_0_ (kV/mm)	α
g-C_3_N_4_/LSR Composites	0 phr	108.2	25.8
	10 phr	97.1	24.3
	20 phr	95.1	22.6
	30 phr	86.1	35.8
	40 phr	79.2	19.1
	50 phr	77.3	15.5
	60 phr	75.1	18.0
O-g-C_3_N_4_/LSR Composites	10 phr	96.4	23.5
	20 phr	94.4	23.2
	30 phr	82.0	19.9
	40 phr	76.5	14.8
	50 phr	70.1	10.0
	60 phr	66.0	10.7

## Data Availability

The original contributions presented in this study are included in the article. Further inquiries can be directed to the corresponding authors.
